# Subacute haematotoxicity after PRRT with ^177^Lu-DOTA-octreotate: prognostic factors, incidence and course

**DOI:** 10.1007/s00259-015-3193-4

**Published:** 2015-09-30

**Authors:** Hendrik Bergsma, Mark W. Konijnenberg, Boen L. R. Kam, Jaap J. M. Teunissen, Peter P. Kooij, Wouter W. de Herder, Gaston J. H. Franssen, Casper H. J. van Eijck, Eric P. Krenning, Dik J. Kwekkeboom

**Affiliations:** Department of Nuclear Medicine, Erasmus University Medical Center, ‘s-Gravendijkwal 230, 3015 CE Rotterdam, The Netherlands; Department of Internal Medicine, Erasmus Medical Center, ‘s-Gravendijkwal 230, 3015 CE Rotterdam, The Netherlands; Department of Surgery, Erasmus Medical Center, ‘s-Gravendijkwal 230, 3015 CE Rotterdam, The Netherlands

**Keywords:** PRRT, ^177^Lu-DOTATATE, Bone marrow, Toxicity, Dosimetry

## Abstract

**Purpose:**

In peptide receptor radionuclide therapy (PRRT), the bone marrow (BM) is one of the dose-limiting organs. The accepted dose limit for BM is 2 Gy, adopted from ^131^I treatment. We investigated the incidence and duration of haematological toxicity and its risk factors in patients treated with PRRT with ^177^Lu-DOTA^0^-Tyr^3^-octreotate (^177^Lu-DOTATATE). Also, absorbed BM dose estimates were evaluated and compared with the accepted 2 Gy dose limit.

**Methods:**

The incidence and duration of grade 3 or 4 haematological toxicity (according to CTCAE v3.0) and risk factors were analysed. Mean BM dose per unit (gigabecquerels) of administered radioactivity was calculated and the correlations between doses to the BM and haematological risk factors were determined.

**Results:**

Haematological toxicity (grade 3/4) occurred in 34 (11 %) of 320 patients. In 15 of the 34 patients, this lasted more than 6 months or blood transfusions were required. Risk factors significantly associated with haematological toxicity were: poor renal function, white blood cell (WBC) count <4.0 × 10^9^/l, age over 70 years, extensive tumour mass and high tumour uptake on the OctreoScan. Previous chemotherapy was not associated. The mean BM dose per administered activity in 23 evaluable patients was 67 ± 7 mGy/GBq, resulting in a mean BM dose of 2 Gy in patients who received four cycles of 7.4 GBq ^177^Lu-DOTATATE. Significant correlations between (cumulative) BM dose and platelet and WBC counts were found in a selected group of patients.

**Conclusion:**

The incidence of subacute haematological toxicity after PRRT with ^177^Lu-DOTATATE is acceptable (11 %). Patients with impaired renal function, low WBC count, extensive tumour mass, high tumour uptake on the OctreoScan and/or advanced age are more likely to develop grade 3/4 haematological toxicity. The BM dose limit of 2 Gy, adopted from ^131^I, seems not to be valid for PRRT with ^177^Lu-DOTATATE.

**Electronic supplementary material:**

The online version of this article (doi:10.1007/s00259-015-3193-4) contains supplementary material, which is available to authorized users.

## Introduction

In the past two decades, peptide receptor radionuclide therapy (PRRT) with radiolabelled somatostatin analogues has been used successfully in patients with somatostatin receptor-positive tumours. One of the most frequently used radiopharmaceuticals is ^177^Lu-DOTA^0^-Tyr^3^-octreotate (^177^Lu-DOTATATE). Patients with neuroendocrine tumours treated with ^177^Lu-DOTATATE have a radiological response rate of 15 – 35 % [1–5]. Generally, PRRT is well tolerated, but the kidneys and bone marrow (BM) are usually the dose-limiting organs.

BM toxicity results from irradiation of and damage to haematopoietic tissue. Grade 3 or 4 haematological toxicity develops in about 5 – 10 % of patients [6–11]. The nadir normally occurs 4 – 6 weeks after each treatment, followed by a recovery phase. The generally accepted threshold dose for radiation-induced BM suppression is 2 Gy, adopted from ^131^I therapy studies [12, 13]. However, up to now, no data have been published that confirm or reject this BM dose limit for ^177^Lu-DOTATATE.

The aim of this study was to analyse short-term haematological toxicity after PRRT with ^177^Lu-DOTATATE. Risk factors analysed included renal function, chemotherapy, baseline cytopenia, tumour mass and patient age. In addition, the individual and mean BM doses were calculated in a subgroup of patients.

## Materials and methods

### Patients

The study included 320 Dutch patients who were treated from January 2000 to December 2007. Inclusion criteria were: patients with neuroendocrine tumour and baseline tumour uptake on [^111^In-DTPA^0^] octreotide scintigraphy (OctreoScan®; Mallinckrodt, Petten, The Netherlands) with accumulation in the tumour at least as high as in normal liver tissue; no prior treatment with PRRT; baseline serum haemoglobin (Hb) ≥6 mmol/l; white blood cell (WBC) count ≥2 × 10^9^/l; platelet (PLT) count ≥ 75 × 10^9^/l; serum creatinine ≤150 µmol/l or creatinine clearance ≥40 ml/min and Karnofsky performance status ≥50. Only Dutch patients were selected, because loss to follow-up is limited in these patients.

This study was part of the ongoing prospective study in patients with neuroendocrine tumours treated with ^177^Lu-octreotate at the Department of Nuclear Medicine, Erasmus University Medical Center Rotterdam. The hospital’s medical ethics committee approved the study. All patients gave written informed consent for participation in the study.

### Treatment

[DOTA^0^,Tyr^3^] octreotate was obtained from BioSynthema (St. Louis, MO). ^177^LuCl_3_ was supplied by IDB-Holland (Baarle-Nassau, The Netherlands) and ^177^Lu-DOTATATE was prepared locally [14].

Granisetron 3 mg or ondansetron 8 mg was injected intravenously 30 min before infusion of ^177^Lu-DOTATATE. Infusion of amino acids (2.5 % arginine and 2.5 % lysine, 1 l) was started 30 min before administration of the radiopharmaceutical and lasted for 4 h. The radiopharmaceutical was coadministered for 30 min using a second pump system. Cycle dosages of 1.85 GBq (50 mCi) were given in 4 patients, 3.7 GBq (100 mCi) in 13 patients, 5.6 GBq (150 mCi) in 14 patients, and 7.4 GBq (200 mCi) in the remaining patients, injected over 30 min. The interval between treatments was 6 – 16 weeks. The intended cumulative dose was 29.6 GBq (800 mCi). Median cumulative activity was 29.6 GBq, range 7.4 – 29.6 GBq. However, the dose was lowered if the calculated kidney dose was higher than 23 Gy. Other reasons for dose reduction or cessation of further therapy were recurrent grade 3 or 4 haematological toxicity and persistent low blood counts.

### Dosimetry

Biodistribution and dosimetry studies were performed in three subgroups of patients. The data on estimated BM doses have been published previously [14–16]. Only patients meeting the inclusion criteria, as stated above, and with complete datasets for dosimetry were included in the present analysis. The BM dose (*D*_rm_) is derived from three sources: (1) from the blood circulating through the marrow cavities (rm), (2) from large organs and tumours with high radioactivity uptake (h), and (3) from the general distribution of radioactivity throughout the remaining whole body (rb):

$$ {D}_{rm}={\tilde{A}}_{rm}DF\left(rm\leftarrow rm\right)+{\tilde{A}}_hDF\left(rm\leftarrow h\right)+{\tilde{A}}_{rb}DF\left(rm\leftarrow rb\right) $$

where Ã is the cumulative activity and DF are the dose factors for red marrow to red marrow, large organs to red marrow, and remainder of the body to red marrow. The contribution to the BM dose from radioactivity distribution within the remainder was calculated (and corrected) according to the method of Wessels et al. [17].

**Fig. 1:**
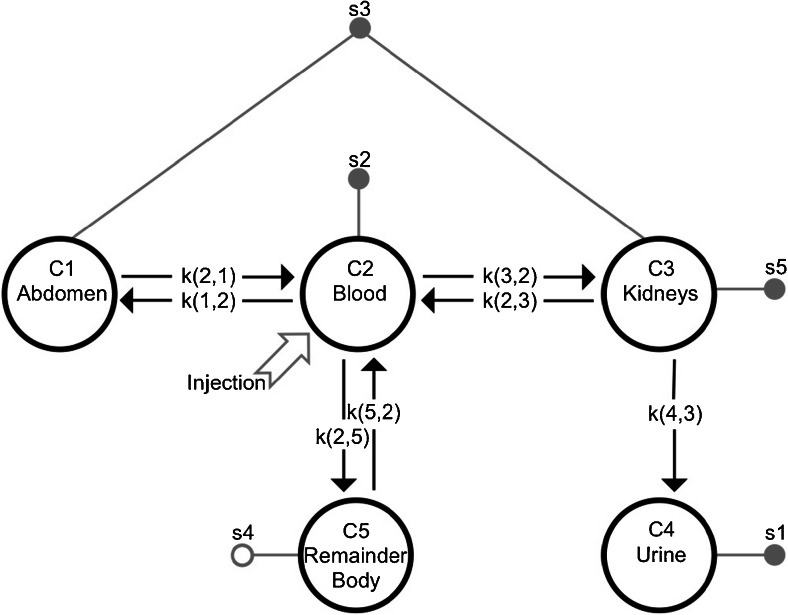
Generalized compartment model for the biodistribution of ^177^Lu-DOTATATE in humans. Compartments (C1 to C5) represent different organs. Flow in both directions between compartments is represented by kinetic transfer components, *k*(*i*,*j*). The *shaded grey circles* represent input (radioactivity) data and the *open grey circle* represents modelled output. Injection is a simulated bolus of ^177^Lu-DOTATATE in the blood compartment

Calculated dose contributions were based on planar scans (at 24, 96 and 168 h after injection), and radioactivity measured in urine (at 1, 6, 24, 48 h after injection) and blood samples (at 0, 10, 30, 60, 90, 120, 360 and 1,440 min after injection). A pharmacokinetics (PK) compartment model was used to describe the biodistribution of radioactivity in organs over time. Organs with physiological uptake (kidneys and abdomen) were added to the central (blood) compartment (Fig. 1). A single compartment linked to the kidney was able to model the urine data. An additional remainder-of-the-body compartment was used to fit the data. Flow in both directions between compartments was modelled by kinetic transfer components,* k*(*i*,*j*). The PK compartment model was numerically solved using SAAMII software (Simulation Analysis and Modeling; SAAM Institute, Seattle, WA). BM dose values were computed using the Olinda/EXM software package (Vanderbilt University) and using dose factors for ^177^Lu [18].

### Toxicity assessment

Haematology, and liver and renal function tests were performed during the 6 weeks before the first therapy, 4 and 6 weeks after each therapy, and at follow-up visits. Haematological toxicity was assessed according to Common Terminology Criteria for Adverse Events (CTCAE v3.0) [19]. This version of CTCAE was used because of well-defined criteria for thrombocytopenia, leucocytopenia and anaemia. Haematological toxicity was modelled for toxicity grade 3/4 in PLT count, WBC count, Hb and a combination of all three. The duration of grade 3/4 haematological toxicity was defined as the time from the last therapy until recovery to toxicity grade 2 or lower.

### Statistical analysis and parameters

SPSS software (SPSS 19; IBM, New York, NY) was used for statistical analysis. Distributions were examined for normality using the Kolmogorov-Smirnov test. Correlations between distributions were evaluated using the *χ*^2^ test, *t* test and analysis of variance. Spearman’s rank correlation coefficient was used for correlation analysis. Regression analysis was performed with the binary logistic model. Conditional step-forward and step-backward methods were used with the following parameters: classification cut-off 0.5, maximum iterations 20, probability for entry 0.05 and removal 0.20. *P* values <0.05 (for both step-forward and step-backward) were considered significant. The following discrete baseline variables were included in the analysis: gender, age over 70 years, presence of bone metastasis, prior chemotherapy, prior external beam radiotherapy, uptake on the OctreoScan, tumour load, chromogranin A >2,000 μg/l, splenectomy, baseline PLT count <150 × 10^9^/l and baseline WBC count <4.0 × 10^9^/l. The creatinine clearance was estimated with the Cockcroft-Gault formula and evaluated as a continuous variable. Similar regression analyses were performed setting thresholds for decreases in PLT count, WBC count and Hb of 15 % and 25 % after the first therapy. Univariate analysis was performed in a subgroup of patients with transient and persistent grade 3/4 haematological toxicity.

In the dosimetric subgroups, the correlations between the percentage reductions in blood cells (Hb, PLT count, WBC count) after the first therapy and dose to the BM were determined using Spearman’s rank correlation coefficients (*r*_S_). The median and mean doses to the BM per unit (gigabecquerels) of administered radioactivity were calculated for each subgroup separately and for all three subgroups combined.

## Results

In total, 324 patients were evaluated. The patient characteristics are summarized in Table 1. Four patients were excluded because of unrelated haematological toxicity (internal bleeding in three and iron-deficiency anaemia in one).

**Table 1:** Baseline characteristics of 320 Dutch patients

Characteristic	Number of patients (%)
Male	164 (51)
Age ≥70 years	62 (19)
Karnofsky performance status ≤70	46 (14)
Elevated chromogranin A	237 (74)
Bone metastasis	72 (23)
Splenectomy	12 (4)
WBC count <4.0 × 10^9^/l at baseline PRRT	16 (5)
Previous therapy	
Chemotherapy	38 (12)
Radiotherapy (external)	32 (10)
Tumour type	
Neuroendocrine	278 (87)
Other	42 (13)
Tumour uptake on baseline OctreoScan	
Equal to or more than normal liver	248 (77)
Higher than kidneys	72 (23)
Tumour mass on baseline OctreoScan	
Equal to or more than normal liver	264 (82)
Higher than kidneys	56 (18)
Cumulative activity (GBq)	
≤22.2	103 (32)
≤29.6	215 (67)
Kidney function, mean (range) creatinine clearance in millilitres per minute, Cockcroft-Gault	99 (35 – 246)

### Toxicity

**Fig. 2:**
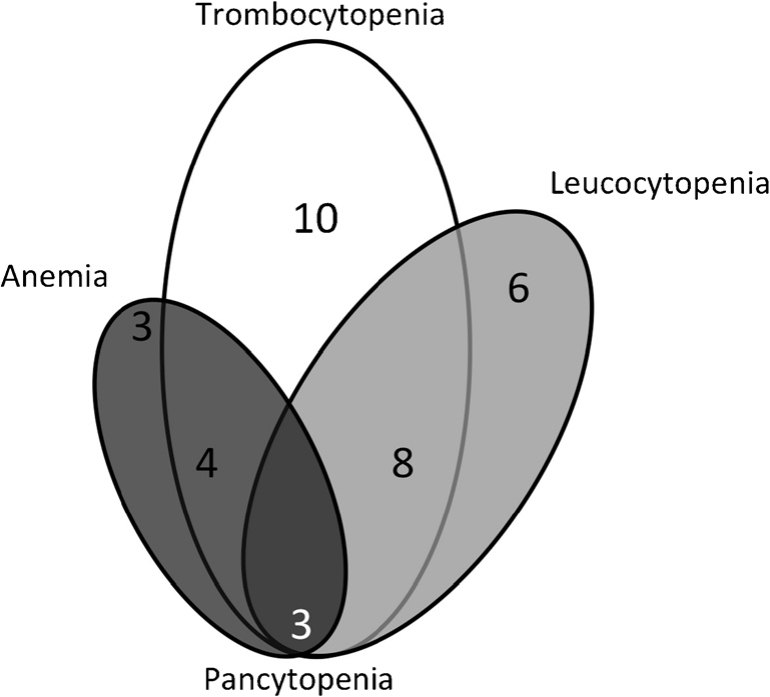
Venn diagram of haematological toxicity (grade 3/4) in 34 out of 320 patients treated with a median cumulative dose of 29.6 GBq ^177^Lu-DOTATATE

Severe subacute haematological toxicity (grade 3/4) occurred 4 to 8 weeks after administration in 34 (11 %) of the 320 patients, with thrombocytopenia in 25 (8 %), leucocytopenia in 17 (5 %), anaemia in 10 (3 %) and pancytopenia (1 %) (Fig. 2).

**Fig. 3:**
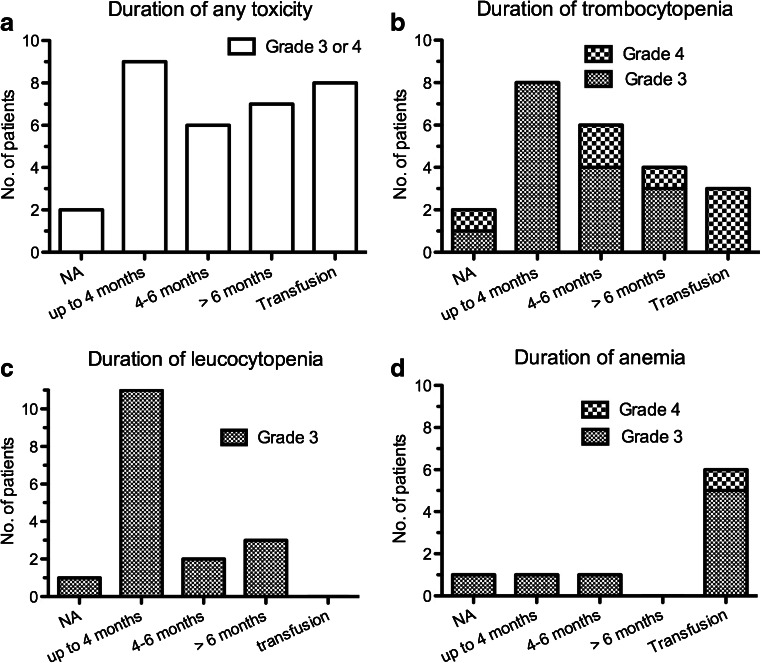
Duration of subacute haematological toxicity (grade 3/4) in 32 of 320 patients treated with a median cumulative dose of 29.6 GBq ^177^Lu-DOTATATE: **a** any toxicity in 32 patients, **b** thrombocytopenia in 23 patients, **c** leucocytopenia in 17 patients, and **d** anaemia in 9 patients (*NA* results not available during follow-up, *Transfusion* patients who received blood cell transfusion after grade 3/4 haematological toxicity. Two patients were excluded (see text)

Two patients were excluded from the analyses for toxicity duration. One patient (with grade 4 thrombocytopenia) died 6 weeks after last the treatment due to bowel obstruction, and one patient (with grade 3 thrombocytopenia and grade 3 anaemia) died 9 weeks after last the treatment due to progressive disease. Of 30 patients, 15 (50 %) had grade 3/4 haematological toxicity lasting more than 6 months or required blood transfusion. The duration of haematological toxicity in these patients is presented in Fig. 3.

Baseline parameters that were significantly associated with grade 3/4 haematological toxicity were: decreased renal function, WBC <4.0 × 10^9^/l, age >70 years, extensive tumour mass, and tumour uptake on the OctreoScan more than uptake in the kidneys (Table 2). No significant association was found for previous chemotherapy.

**Table 2:** Baseline clinical parameters associated with grade 3/4 haematological toxicity in 34 of 320 patients treated with a median cumulative dose of 29.6 GBq ^177^Lu-DOTATATE from logistic regression analysis with the stepwise method (step-forward and step-backward)

Variable	Step-forward		Step-backward	
	Coefficient^b^	*p* value	Coefficient^b^	*p* value
Any toxicity (Hb/PLT/WBC)				
Creatinine clearance (Cockcroft-Gault)^a^	−0.160	0.028	−0.150	0.044
Bone metastasis	1.055	0.017	0.912	0.056
WBC count <4.0 × 10^9^/l at baseline^a^	1.828	0.005	1.741	0.011
Tumour uptake on Octreoscan > kidney uptake	0.867	0.051	1.055	0.023
Previous radiotherapy	Not in predictive equation		1.225	0.074
Previous chemotherapy	Not in predictive equation		1.171	0.161
Haemoglobin				
Age >70 years^a^	1.698	0.045	1.860	0.039
Extensive tumour mass^a^	2.551	0.002	2.570	0.003
Previous radiotherapy	Not in predictive equation		2.165	0.036
Platelets				
Creatinine clearance (Cockcroft-Gault)^a^	−0.022	0.010	−0.025	0.008
Bone metastasis	1.268	0.009		
WBC count <4.0 × 10^9^/l at baseline	1.731	0.016	1.565	0.196
Extensive tumour mass	Not in predictive equation		1.174	0.024
Previous radiotherapy	Not in predictive equation		1.392	0.055
Previous chemotherapy	Not in predictive equation		−1.604	0.144
White blood cells				
Age >70 years	Not in predictive equation		1.161	0.062
WBC count <4.0 × 10^9^/l at baseline^a^	2.436	0.001	2.531	0.000
Tumour uptake on Octreoscan > kidney uptake^a^	1.321	0.022	1.549	0.010
Previous radiotherapy	1.363	0.068	Not in predictive equation	

^a^Variable statistically significant (*p* < 0.05) in multivariate analyses

^b^Logistic coefficient in predictive equation

Of 30 patients with persistent (more than 6 months) haematological toxicity or who required blood transfusions, 15 had significantly more tumour mass on the baseline OctreoScan than patients with transient (6 months or less) grade 3/4 haematological toxicity. No significant difference in other baseline variables was found between these two subgroups (data not shown). In patients with decrease in Hb of more than 15 % after the first therapy, previous radiotherapy was an additional significant factor in the logistic regression analysis (*p* = 0.005 and *p* = 0.001 for step-forward and step-backward methods, respectively). In patients with a decrease in Hb, PLT count and/or WBC count of more than 25 % after the first therapy, decreased renal function at baseline was the only significant variable (*p* < 0.05).

### Dosimetry

The dosimetry analysis included 32 patients split into three groups with different cycle doses (1.85, 3.7 and 7.3 GBq ^177^Lu-DOTATATE). Of the 32 patients, 25 patients were treated according protocol; in two patients no complete dosimetric data were available. The BM dose after the first therapy was determined in 25 patients: 4 patients in group 1 (cycle dose 1.85 GBq), 7 patients in group 2 (cycle dose 3.7 GBq) and 14 patients in group 3 (cycle dose 7.3 GBq). The cumulative dose was 14.8 GBq in 1 patient, 22.2 GBq in 9 patients and 29.6 GBq in 15 patients. The median dose (and range) to the BM per unit of administered radioactivity in patients in group 1 and group 2 was 69 mGy/GBq (54 – 73 mGy/GBq) and 75 mGy/GBq (35 – 139 mGy/GBq), respectively. In group 3 the median dose (and range) was 51 mGy/GBq (24 – 116 mGy/GBq) excluding one outlier of 331 mGy/GBq. In this patient the urinary excretion data could not be fitted correctly in the compartment model. This resulted in a long residence time of activity in the total body and in an exceptionally high BM dose, leading to the exclusion of this patient from further analysis (Supplementary Data). Despite a high calculated BM dose, this patient did not develop grade 3 or 4 haematological toxicity.

**Fig. 4:**
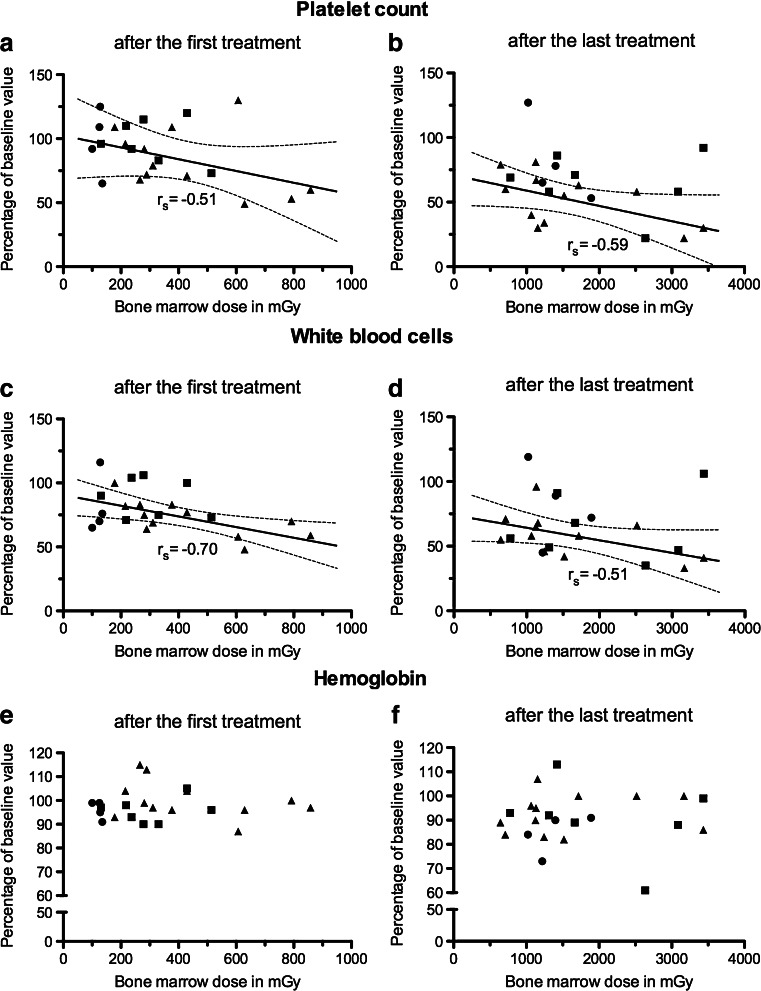
Platelet counts (**a**, **b**), white blood cell counts (**c**, **d**) and haemoglobin (**e**, **f**) expressed as percentages of the baseline values in relation to bone marrow dose in 23 patients after the first and last treatments with ^177^Lu-DOTATATE (*circles* group-1, 1.85 GBq, *n* = 4; *squares* group 2, 3.70 GBq, *n* = 7; *triangles* group 3, 7.40 GBq, *n* = 12). *Solid lines* is linear regression with 95 % confidence intervals (*dotted lines*). Significant Spearman’s rank correlation coefficients with (one-tailed) *P* values in group 3: **a** *r*_S_ = −0.51 with *P* < 0.05, **b** *r*_S_ = −0.59 with *P* = 0.02, **c** *r*_S_ = −0.70 with *P* = 0.01, **d** *r*_S_ = −0.51 with *P* < 0.05

Data from groups 1, 2 and 3 combined were normally distributed (Kolmogorov-Smirnov test) allowing calculation of the mean BM dose. The mean BM dose (excluding one outlier) per unit of administered activity in the 24 evaluable patients was 0.067 ± 0.007 mGy/MBq. At an activity administration schedule of 4 × 7.4 GBq (which most patients received) this would lead to a BM dose of 2.0 ± 0.2 mGy. Three (13 %) of 23 patients developed grade 3/4 haematological toxicity. No significant difference in BM dose was observed between these 3 patients and the other 20. Significant Spearman’s rank correlation coefficients and *P* values (one-tailed) were found in group 3 between (cumulative) BM dose and PLT count after the first and last treatments (*r*_S_ = −0.51 with *P* < 0.05 and *r*_S_ = −0.59 with *P* = 0.02, respectively) and WBC count (*r*_S_ = −0.70 with *P* = 0.01 and *r*_S_ = −0.51 with *P* < 0.05, respectively; Fig. 4). No significant correlation between (cumulative) BM dose and haemoglobin was found in group 3, and no significant correlations were found between (cumulative) BM dose and blood cells in group 1 and group 2.

## Discussion

Subacute haematological grade 3/4 toxicity was observed in 34 (11 %) of 320 patients receiving ^177^Lu-DOTATATE. In half of these patients, toxicity persisted for more than 6 months or blood transfusions were required. This is in accordance with data from other groups [5–10]. Long-term haematological toxicities, such as myelodysplastic syndrome (MDS) and acute leukaemia (AL), have been found in patients receiving PRRT with ^177^Lu-DOTATATE [6, 11]. Also in our patient group, MDS and AL were observed, but since these events have a rare complex stochastic character, they will be reported in a separate study. In a recent study, long-term side effects of PRRT with ^90^Y-octreotide and/or ^177^Lu-octreotate were investigated [6]. The authors found more haematological toxicity after PRRT in patients with baseline nephrotoxicity (transient or persistent elevation in creatinine). The prolonged circulation time of ^177^Lu-DOTATATE in patients with a poor renal function is probably the most important factor that explains the increased toxicity to the BM, as shown by Svensson et al. [20]. Also anaemia is common in these patients due to a reduction in renal erythropoietin production. In our study, poor renal function was also found to be a predictor of haematological toxicity. We also found that a low baseline WBC count is a predictor of grade 3/4 haematological toxicity. This is in line with the findings of a recent study showing that baseline cytopenia is a predictor of haematological toxicity after PRRT [9].

From a dosimetric point of view, it has been theorized that patients with a large tumour burden and high receptor density have lower amounts of circulating activity [21]. Therefore the radiation to normal tissues could be less than in patients with a low tumour burden. However, in our study, patients with more tumour mass at baseline were significantly more likely to have grade 3/4 haematological toxicity. Furthermore, high tumour burden was more frequently found in patients with persistent grade 3/4 haematological or who required blood transfusion. This suggests that tumour burden plays a role in the development and duration of haematological toxicity, in contrast to the recently described tumour sink effect [22]. That study showed that internalization of ^68^Ga-DOTATATE in the tumour leads to a significant decrease in uptake in healthy tissue, the so-called tumour sink effect [22]. The authors extrapolated their results and speculated on a similar effect for PRRT. However, the main contributing factor for radiation dose to an organ (i.e. the BM) from PRRT is the exposure to radiation over time and the organ dose cannot be based on a distribution with only one time point. In our limited subgroup of patients in whom biodistribution and dosimetry data were available, we were not able to demonstrate the tumour sink effect.

Past chemotherapy was not a clear risk factor in our analyses. This finding can be explained by the limited number of patients who received chemotherapy in our series (38 of 320 patients, 12 %). In other studies more than 25 % of patients have had a history of chemotherapy [6, 8, 9]. In particular, chemotherapy regimens with alkylating agents (e.g. cisplatin, carboplatin, oxaliplatin) or topoisomerase II inhibitors (e.g. etoposide) can damage the DNA of haematopoietic cells [23]. A small number (12 %) of patients in our study had received this type of chemotherapy in the past, therefore limiting the statistical power of this (possible) risk factor.

A potential protective effect of splenectomy on the development of haematological toxicity in patients receiving ^177^Lu-DOTATATE has recently been reported [9]. The spleen is a major reservoir of blood cells and uptake of radioactivity is mainly caused by the presence of somatostatin receptors on lymphocytes [14, 24]. Blood cells circulating throughout the spleen may be damaged leading to a reduction in peripheral blood cell counts. However, in another clinical study with ^177^Lu-DOTATATE/DOTATOC, no correlation was found between dose to the spleen and haematological toxicity during PRRT [25]. In our study, none of the 12 patients who had had a splenectomy developed grade 3/4 haematological toxicity. This supports the idea that splenectomy has a protective effect in patients receiving PRRT, but statistical analysis could not be performed due to the limited number of patients with splenectomy.

PRRT using ^177^Lu-DOTATATE shows similarities to ^131^I treatment, because of the comparable half-life (6.7 and 8.0 days, respectively) and similar energies of the emitted β radiation (with average energies of 133 and 182 keV, respectively). Therefore in 2000, we accepted a maximum BM dose for PRRT with ^177^Lu-DOTATATE, which was adopted from clinical studies with ^131^I treatments. The upper BM dose limit was set to 2 Gy, based on early work in thyroid cancer patients treated with ^131^I [12]. In that study, 122 doses of ^131^I were administered to 59 patients with metastasized thyroid cancer. In 14 administrations of ^131^I, serious radiation complications were observed (Table 3, original table). The authors stated that serious radiation complications per treatment cycle were more frequent when the total dose to the blood exceeded 200 rad (2 Gy) with a significance of *P* = 0.03 (Table 3). However, only 8 of 14 serious radiation complications were related to the BM; the other complications were pneumonitis or vomiting. When we repeated the analysis, taking into account only serious BM complications (Table 3, modified table), no significantly higher frequency of haematological toxicity for BM radiation doses more than 2 Gy could be demonstrated (Fisher’s exact test, *P* = 0.68). Also, all eight patients with radiation complications (related to the BM) had metastatic disease to the bone. Bone metastases are a source of radiation after PRRT and could contribute to an increase in BM dose. However, in our multivariate analysis, the presence of bone metastases was not a risk factor for developing grade 3/4 haematological toxicity.

**Table 3:** Complications in relation to total BM radiation dose reported by Benua et al. [12], and with new modifications. Data were derived from patients treated with radioiodine ^131^I treatment

Blood total radiation (Gy)	No. of doses	Original table^a^			Modified table^b^		
		Radiation complications			BM radiation complications		
		Severe	Fatal	Total in percent	Severe	Fatal	Total in percent
0 – 0.99	5	0	0	0	0	0	0
1.00 – 1.99^c^	24	1	0	4	1	0	4
2.00 – 2.99	33	5	1	18	3	1	12
3.00 – 3.99	7	1	1	29	1	0	14
4.00 – 4.99	9	0	2	22	0	1	11
Over 5.00	7	2	0	29	1	0	14
Unknown	37	1	0	3	0	0	0
Total	122	10	4	7	6	2	7

^a^Original table of Benua et al. [12]

^b^Modified table with only serious bone marrow radiation complications

^c^Significantly more frequent complications with total dose >200 rad are stated in the original table, but are not significant in the modified table

Another comment on the study by Benua et al. is that the radiation complications per unit administered dose were analysed and the cumulative BM dose per patient was not considered. This resulted in a double count of radiation complications in one patient (after the first and second dose of ^131^I). In our study, haematological toxicity was counted only once per patient since the chance of recurrent haematological toxicity in one patient is relatively high. We also analysed the cumulative BM dose instead of complications per administration, considering that multiple sequential treatments reduce the BM reserve. In a more recent article, the BM limit was set to 3 Gy for ^131^I treatment, based on 104 treatments in 83 thyroid cancer patients [26]. No permanent BM suppression was observed, but two patients required PLT and red blood cell transfusion because of pancytopenia.

In the past decade, several studies with BM dose estimates using ^177^Lu-DOTATATE have been reported (Table 4). Variations in the reported BM dose estimates can partly be attributed to differences in accuracy of dosimetric methods [27]. In a recent Swedish study, 200 patients who were treated with 7.4 GBq ^177^Lu-DOTATATE were analysed and BM doses were calculated based on blood-based and organ-based analysis of the whole-body images. The authors calculated a maximum BM dose of 0.4 Gy per cycle of 7.4 GBq, which would result in a cumulative BM dose of 1.6 Gy for four cycles. Our data showed an estimated mean BM dose of 2.0 Gy (SD 0.2 Gy) in 184 out of 320 patients who received four cycles of 7.4 GBq ^177^Lu-DOTATATE. Therefore, half of these patients (92 of 184) received a BM dose of more than 2 Gy. If the true maximum tolerated dose to the BM were 2 Gy, these 92 patients would theoretically be more prone to develop haematological toxicity. However, we found haematological toxicity in only 34 of the 320 patients. This supports the idea that a higher BM dose limit for PRRT with ^177^Lu-DOTATATE is appropriate. Another argument for a different BM dose maximum is the success of retreatment with extra cycles of PRRT without serious haematological side effects [28]. In our analysis of this type of retreatment, in which selected patients received a cumulative BM dose of approximately 3 Gy, only 5 (16 %) of 32 patients developed grade 3/4 haematological toxicity after two additional cycles of ^177^Lu-DOTATATE. In another study reversible haematotoxicity (grade 3/4) was found in 7 (21.2 %) of 33 patients who underwent salvage PRRT [29]. In our ongoing study in Erasmus MC, we have treated a selected group of patients with multiple additional cycles of ^177^Lu-DOTATATE with cumulative doses up to 59.2 GBq resulting in an estimated mean BM dose of more than 3 Gy with limited haematological toxicity (unpublished data).

**Table 4:** Overview of reported data on BM dosimetry for PRRT with ^177^Lu-DOTATATE

Reference	Number of patients	Dosimetric method	Administered activity (GBq)	Amino acids	BM dose	
					Per unit administered activity (Gy/GBq)	For four cycles of 7.4 GBq (Gy)
14	5	Planar	1.85	Lys/Arg	0.070 ± 0.009	2.1
36	69	Planar	3 – 7	Lys/Arg	0.050 ± 0.020	1.5
16	13	Planar	7.47	Lys/Arg	0.01 – 0.13	0.30 – 3.85
4	16	SPECT/CT	7.4	Vamin 14	0.070 ± 0.020	2.1
2	12	Not reported	3.7 – 7.4	Lys	0.002 – 0.060	0.6 – 1.8
37	200	SPECT/CT	7.4	Vamin 14	0.006 – 0.050	0.2 – 1.5
This study	25^a^	Planar	1.85 – 7.3	Lys/Arg	0.067 ± 0.007	2.0

*Lys*/*Arg* lysine 2.5 %/arginine 2.5 %, *Lys* lysine 2.5 %

^a^Of 320 patients

Several groups have investigated the role of BM dosimetry in radionuclide treatments for predicting haematological toxicity. A weak negative correlation (*r*_p_ = −0.47) between neutrophils at nadir and measured whole-body absorbed dose was found in 20 patients treated with ^131^I-MIBG [30]. In a PRRT study with ^90^Y-DOTATOC, a correlation (*R* = 0.58) was found between BM absorbed dose and PLT count reduction at nadir [31, 32]. BM dose was calculated in 12 patients based on plasma samples, assuming that the activity concentration in the BM was equal to that in the plasma. ^86^Y-DOTATOC PET was performed after therapy and showed uptake in the vertebrae. Taking the radioactivity in the spine into the dosimetric calculations, a better correlation (*R* = 0.82) was found between BM absorbed dose and PLT count reduction at nadir. However, 24 patients did not demonstrate sufficient uptake of ^86^Y-DOTATOC in the spine to provide usable BM dose measurements. In our study, we found similar correlation coefficients between the relative decrease in blood cells and (cumulative) radiation dose to the BM. We found no correlation between BM dose and Hb, which can be explained by minimal effects on circulating erythrocytes after 2 Gy of irradiation [33]. These weak correlations between decreases in blood count and BM dose indicate that current dosimetry cannot fully predict haematological toxicity. Additional clinical factors have to be taken into account to predict haematological toxicity in PRRT.

Further research should focus on reporting BM dose in patients receiving PRRT with ^177^Lu-DOTATATE. BM dose limits should be explored at the population level with clinical toxicity grading (e.g. CTCAE) as outcome. However, current BM dosimetry is imprecise and varies due to differences in method of BM dose calculation [34]. Also the absorbed BM dose does not reflect the damage done to the haematopoietic stem cell department. If BM dosimetry can reflect the actual dose to haematopoietic stem cells, it will have a more prominent place in clinical practice during PRRT. In vivo markers might also be an option for assessment of BM status after irradiation with PRRT. In a recent article, γ-H2AX foci in lymphocytes were successfully used for monitoring ionizing radiation-induced DNA double-strand breaks in patients treated with ^177^Lu-DOTATATE [35]. However, the response of γ-H2AX foci varied significantly between patients over time, making it less suitable for individual monitoring. In future, BM radiation dose could provide information for decision-making in a clinical setting, but at present BM dosimetry plays a minor role in clinical practice. Clinical parameters and blood cell count recovery are currently the most important criteria for individual PRRT planning.

### Conclusion

The prevalence of subacute haematological toxicity (grade 3 or 4) after PRRT with ^177^Lu-DOTATATE is low (11 %). Our dosimetric calculations of the absorbed BM dose support the idea that a BM dose limit of more than 2 Gy seems appropriate for PRRT with ^177^Lu-DOTATATE. A correlation was found between BM dose and decreases in blood counts, but clinical risk factors are currently the most important parameters for prediction of clinical toxicity. Patients with impaired renal function, low WBC count, extensive tumour mass, high tumour uptake on the OctreoScan and/or those of advanced age are more likely to develop grade 3 or 4 haematological toxicity. Our data support the idea that a higher BM dose limit of 2 Gy is appropriate for PRRT with ^177^Lu-DOTATATE.

## Electronic supplementary material

ESM 1 (DOCX 738 kb)
