# Supplementary material for: Subacute haematotoxicity after PRRT with 177Lu-DOTA-octreotate: prognostic factors, incidence and course
Source: Eur J Nucl Med Mol Imaging. 2015 Sep 30;43:453–63. doi: 10.1007/s00259-015-3193-4 (PMC4731438; doi:10.1007/s00259-015-3193-4)
Supplement: Supplementary file 1 — (DOCX 738 kb) [file 259_2015_3193_MOESM1_ESM.docx]

**Supplemental Data**

**Subacute hematotoxicity after PRRT with ^177^Lu-DOTA-Octreotate: Prognostic factors, incidence and course**

Hendrik Bergsma^1^, Mark Konijnenberg^1^, Boen L.R. Kam^1^, Jaap J.M. Teunissen^1^, Peter P. Kooij^1^, Wouter W. de Herder^2^, Gaston J.H. Franssen^3^, Casper H.J. van Eijck^3^, Eric P. Krenning^1^ and Dik J. Kwekkeboom^1^;

Departments of ^1^ Nuclear Medicine, ^2^ Internal Medicine and ^3^ Surgery, Erasmus Medical Center, ‘s-Gravendijkwal 230, 3015 CE Rotterdam, The Netherlands

For correspondence or reprints contact: Hendrik Bergsma MD, Department of Nuclear Medicine, ErasmusMC, Department of Nuclear Medicine, Erasmus University Medical Center Rotterdam, 's Gravendijkwal 230, 3015 CE Rotterdam - The Netherlands. E-mail: bergsmahb@gmail.com, Telephone: +31 10 704 0132, Fax: +31 10 703 4647

**Methods**

*Dosimetry*

The method used for calculating the bone marrow (BM) radiation dose takes into account the radiation from β-rays of 177Lu-DOTATATE in the blood circulating through the trabecular bone, and from penetrating γ-rays from radioactivity dispersed throughout the remainder of the body.The absorbed dose (D) to red marrow (rm) can be estimated using the MIRD schema. Contributions to the BM dose are from localized in the marrow tissues (self-dose) and in the remainder tissues (rm) of the body (cross-dose):

$$D_{rm}=D_{rm}^{Self}+D_{rm}^{Cross}$$

$$D_{rm}=Ã_{rm}S\left( rm\leftarrow rm \right)+{(Ã}_{WB}-Ã_{rm})S(rm\leftarrow rb)$$

where Ã is the cumulated activity and S are the S factor for red marrow to red marrow and the remainder of the body to red marrow.

Our BM dose (D_rm_) is derived from three source contributions: (1) from the blood circulating through the marrow cavities (rm), (2) from large organs and tumours with high radioactivity uptake (h) and (3) from the general distribution of radioactivity throughout the remaining whole body (rb):

$$D_{rm}=Ã_{rm}DF\left( rm\leftarrow rm \right)+\sum_{h} Ã_{h}DF(rm\leftarrow h)+Ã_{rb}DF(rm\leftarrow rb)$$

where Ã is the cumulated activity and DF are the dose factors for red marrow to red marrow, large organs to red marrow and remainder of the body to red marrow. Contribution to the bone marrow dose from radioactivity distribution within the remainder were calculated according to the method by Wessels et al. [1]. Radioactivity in the bone marrow first follows the plasma perfusion through the marrow space and at later times will be according to the whole body distribution. Both contributions have been taken into account in accordance with the bone marrow dosimetry performed by F Forrer *et al.* [2], which data are included in the present data set.

**Results**

*Incomplete dosimetric DATA*

Incomplete 1, Patient (Po****)

In this patient no data for urinary clearance was available, therefore it was not possible to perform bone marrow dose calculation using the compartment model.


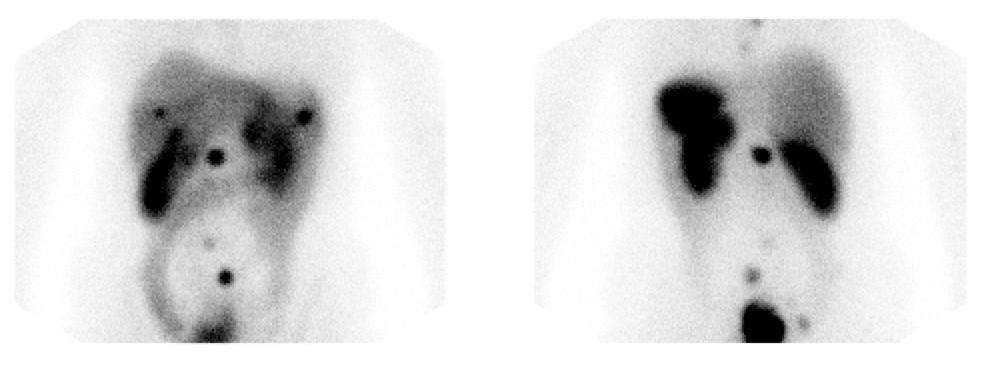


B

A

Posterior

Anterior

Fig 1. Anterior (A) and posterior (B) planar scans, 24 hr after the 1^st^ therapy of patient Po****.

Incomplete 2, Patient (Me****)

In this patient no blood radioactivity data were available. Consequently no compartment modelling was possible and numerical fitting was performed to obtain the cumulated activity in the bowel and the remainder of the body. The uptake in the bowel was exceptionally high, leading to a residence time of 79 h. As only 41% of the activity was cumulatively excreted in the urine at 48 h this lead to a total body residence time of 135 h. Using the dose conversion factor DF (BM←remainder) = 2.67 E-7 mGy/MBq.s from Olinda, this would lead to a bone marrow dose of 0.13 mGy/MBq.


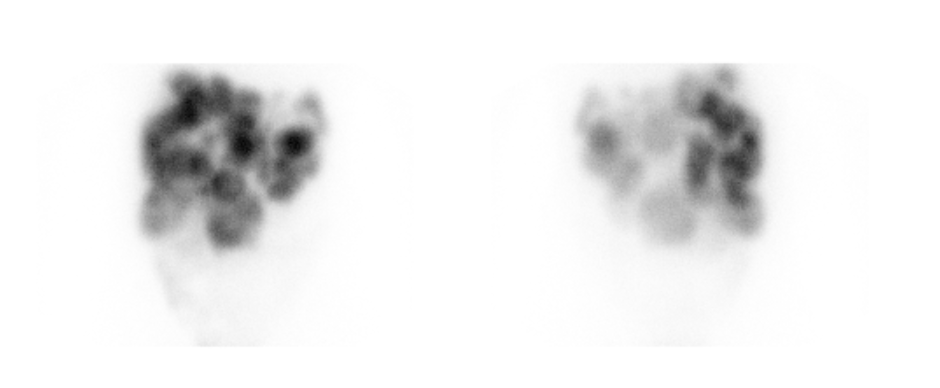


Posterior

Anterior

B

A

Fig 1. Anterior (A) and posterior (B) planar scans, 24 hr after the 1^st^ therapy of patient Me****.


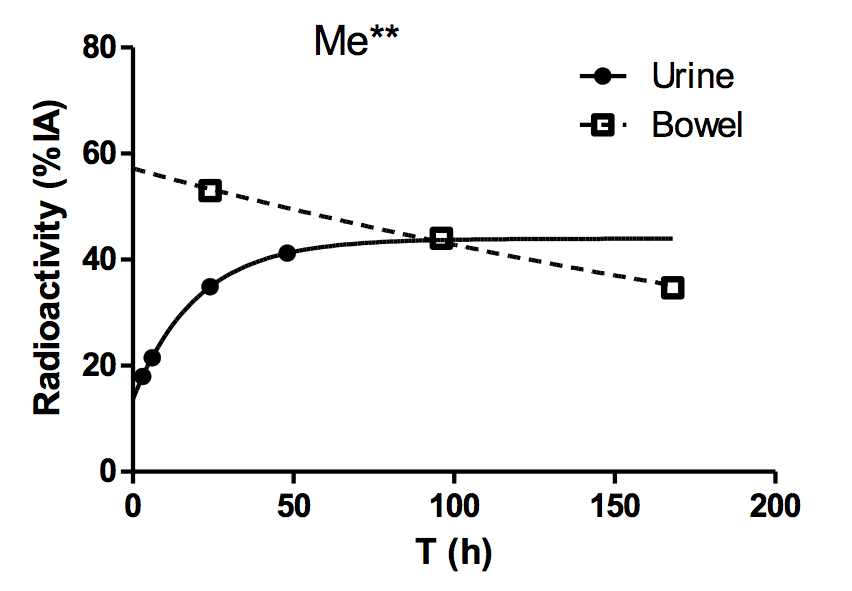


Fig 2. Time-activity curves for urinary clearance and total bowel uptake of ^177^Lu-Dotatate in patient Me****. Only 44% (95%CI: 42-45%) of the radioactivity is measured in the cumulative urinary clearance and it shows a half-life of 14 h (12-16 h). The 57% (43-71%) uptake in the bowel clears with 239 h (127 – 2183 h) half-life.

*Outlier*

Outlier, Patient (Ko****)

The compartmental model failed to fit the urinary excretion data of patient Ko*** correctly. The clearance from the bowel was slow with final clearance half-life of 290 h, leading to a residence time of 33 h. The total body residence time is 72 h, based on the numerical fit, this leads to a bone marrow dose of 69 μGy/MBq. This value is 5σ above the mean value of 14 ± 10 μGy/MBq. In this case the blood time-activity curve determines the bone marrow residence time to be 1.0 h and the contribution to the bone marrow dose from the remainder needs to be corrected according to the method by Wessels et al. [1]. This leads to a corrected bone marrow dose by the remainder of 3.1 μGy/MBq, which is still 5σ above the corrected mean value of 0.67 ± 0.46 μGy/MBq.


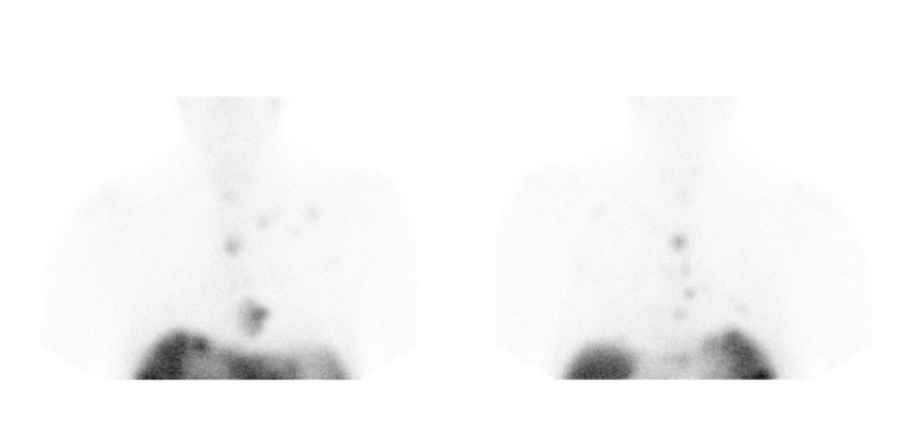


B

A

D

C


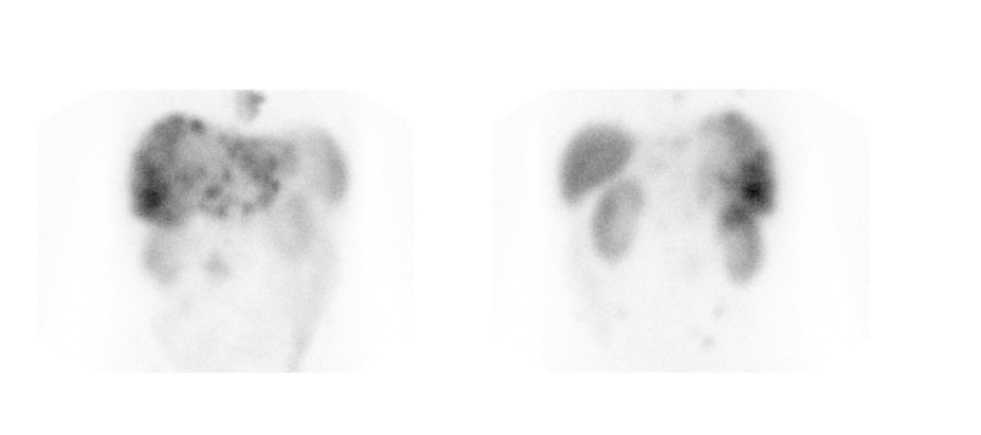


Posterior

Anterior

Fig 1. Anterior (A) and posterior (B) planar scans, 24 hr after the 1^st^ therapy of patient Kon****-2.


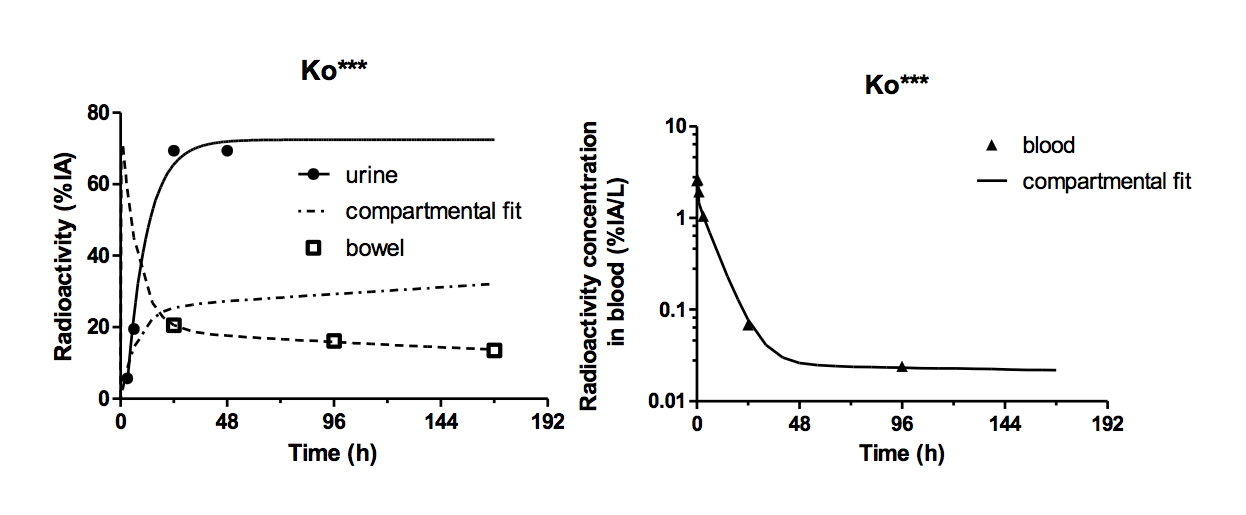


Figure 3. Time-activity curves for urine, bowel and blood activity in patient Ko***. The curves through the data are the result of the compartmental fitting, except for through the urine data, where a single-exponential build-up curve was fitted numerically instead.

**References**

1. Wessels BW, Bolch WE, Bouchet LG, Breitz HB, DeNardo GL, Meredith RF et al. Bone marrow dosimetry using blood-based models for radiolabeled antibody therapy: a multiinstitutional comparison. Journal of Nuclear Medicine. 2004;45(10):1725-33.

2. Forrer F, Krenning EP, Kooij PP, Bernard BF, Konijnenberg M, Bakker WH et al. Bone marrow dosimetry in peptide receptor radionuclide therapy with [177Lu-DOTA(0),Tyr(3)]octreotate. European journal of nuclear medicine and molecular imaging. 2009;36(7):1138-46. doi:10.1007/s00259-009-1072-6.
